# Primary prevention cardiovascular disease risk prediction model for contemporary Chinese (1°P-CARDIAC): Model derivation and validation using a hybrid statistical and machine-learning approach

**DOI:** 10.1371/journal.pone.0322419

**Published:** 2025-07-28

**Authors:** Yekai Zhou, Celia Jiaxi Lin, Qiuyan Yu, Joseph Edgar Blais, Eric Yuk Fai Wan, Emmanuel Wong, Kathryn Tan, David Chung-Wah Siu, Kai Hang Yiu, Esther Wai Yin Chan, Doris Yu, William Wong, Tak-Wah Lam, Ian Chi Kei Wong, Ruibang Luo, Celine S. L. Chui

**Affiliations:** 1 School of Computing and Data Science, The University of Hong Kong, Hong Kong Special Administration Region, China; 2 Laboratory of Data Discovery for Health (D^2^4H), Hong Kong Science Park, Hong Kong Science and Technology Park, Hong Kong Special Administration Region, China; 3 School of Nursing, The University of Hong Kong, Hong Kong Special Administration Region, China; 4 Centre for Safe Medication Practice and Research, Department of Pharmacology and Pharmacy, The University of Hong Kong, Hong Kong Special Administration Region, China; 5 Department of Family Medicine and Primary Care, School of Clinical Medicine, Li Ka Shing Faculty of Medicine, The University of Hong Kong, Queen Mary Hospital, Hong Kong Special Administration Region, China; 6 Advanced Data Analytics for Medical Science (ADAMS) Limited, Hong Kong Special Administration Region, China,; 7 Department of Medicine, School of Clinical Medicine, Li Ka Shing Faculty of Medicine, The University of Hong Kong, Hong Kong Special Administration Region, China; 8 Aston Pharmacy School, Aston University, Birmingham, United Kingdom; Cedars-Sinai Heart Institute, UNITED STATES OF AMERICA

## Abstract

**Background:**

Cardiovascular disease (CVD) is the leading cause of mortality and morbidity in China and worldwide while we are lacking in validated primary prevention model specifically for Chinese. To identify CVD high-risk individuals for early intervention, we created and validated a primary prevention risk prediction model, Personalized CARdiovascular DIsease risk Assessment for Chinese (1°P-CARDIAC), in contemporary Chinese cohorts in Hong Kong.

**Methods:**

Patients without any history of CVD was categorized as derivation and validation cohorts based on their different geographical location of residence in Hong Kong. The outcome was the first diagnosis of a composite of coronary heart disease, ischemic or hemorrhagic stroke, peripheral artery disease, and revascularization. The full model incorporated all available variables in the dataset as clinical laboratory tests, disease and medication history, family history of disease, demographic factors, and healthcare utilization. We employed XGBoost Cox model and multivariate imputation with chained equation (MICE) for derivation and missing data replacement. A basic model was developed with the integration of statistically significant and important subset of risk variables by least absolute shrinkage and selection operator (LASSO) regression. Validation was performed by 1000 bootstrap replicates and compared to four existing models: PREDICT, pooled cohort equation (PCE), China-PAR, and Framingham (Asian).

**Results:**

The study included 179,953 patients in the derivation cohort and 1,083,924 patients across two independent validation cohorts. A total of 103 covariates were included in the full model whilst 8 covariates were included the basic model. It demonstrated good performance with C-statistic of 0.87 (95% CI: 0.87, 0.87), calibration slope of 0.94 in the full model. The C-statistic in the basic model was 0.75 (95% CI: 0.75, 0.75) with calibration slope of 0.91. Other comparison risk models have lower C statistic ranging from 0.68 to 0.72.

**Conclusion:**

We developed and validated 1°P-CARDIAC, a CVD risk prediction model for primary prevention applying a novel hybrid statistical and machine-learning approach. Validation results suggest that it may offer improved performance compared to commonly used risk models. The 1°P-CARDIAC yields the similar level of accuracy and performance between basic and full model. It demonstrated both effectiveness and versatility in harnessing the power of big data and which has the potential to serve as a promising method for CVD primary prevention and improving public health outcome.

## 1. Introduction

Prevention is especially important since cardiovascular disease (CVD) is one of the primary causes of mortality and morbidity in China [1,2] and worldwide [3]. The rapid development in China during the 1980s and 1990s has led to notable shifts in CVD-related trends [4]. A cost-effective way to prevent CVD is to identify people at a higher risk of CVD with risk scores and provide early interventions [5]. Despite having many CVD risk assessment tools for primary prevention, there are very few well validated ones available for Chinese [6]. Established CVD risk scores like The Framingham Heart Study aim to enhance CVD prognosis and prevention [7]. However, most of these risk scores were developed based on Western populations. Although a few risk scores have been created using Chinese cohorts [8–10], these were based on cohort studies established in a simply point scale system in the past decades. Particularly, those popular models, such as China-PAR and Framingham Asia, were not fully account for recent changes in lifestyle, diet, economic development, and healthcare resources between different districts in China. CVD is a multifaceted condition that interacts with other health issues such as diabetes mellitus (DM) [11]. In that case, machine-learning is a well-suited for complex relationship handling which traditional statistical methods were often oversimplify [12]. Its high dimensionality on CVD risk prediction enable to involves numerous variables consideration during the predictors selection process [13]. With the advancement of machine-learning technology and availability of longitudinal electronic health records (EHR), there is an opportunity to incorporate a wide range of parameters in estimating CVD risk. Therefore, there is a need for a CVD risk assessment tool for primary prevention, considering wide array of parameters, to identify high-risk patients in a contemporary Chinese population.

In this study, we developed and validated a primary prevention model for Personalized CARdiovascular DIsease risk Assessment for Chinese (1°P-CARDIAC) for contemporary Chinese cohorts in Hong Kong. Although the medical research has embraced numerous revolutions in the era of big data [14–20], current CVD prediction methods are still limited by traditional statistical approaches and a restricted set of variables. We developed and utilized a novel hybrid statistical machine-learning model to leverage the potential of big data for better CVD prediction. To evaluate its effectiveness, we compared the performance of 1°P-CARDIAC with several representative tools – i.e., PREDICT [21], the 2013 American College of Cardiology/American Heart Association Pooled Cohort Equations (PCE) [22], Prediction for ASCVD Risk in China (China-PAR) [9], and Low-information cardiovascular risk prediction equations from the Asian and the Framingham cohorts (Framingham [Asian]) [23]– across three study cohorts in Hong Kong.

## 2. Method

### a. Study cohorts

**Fig 1:**
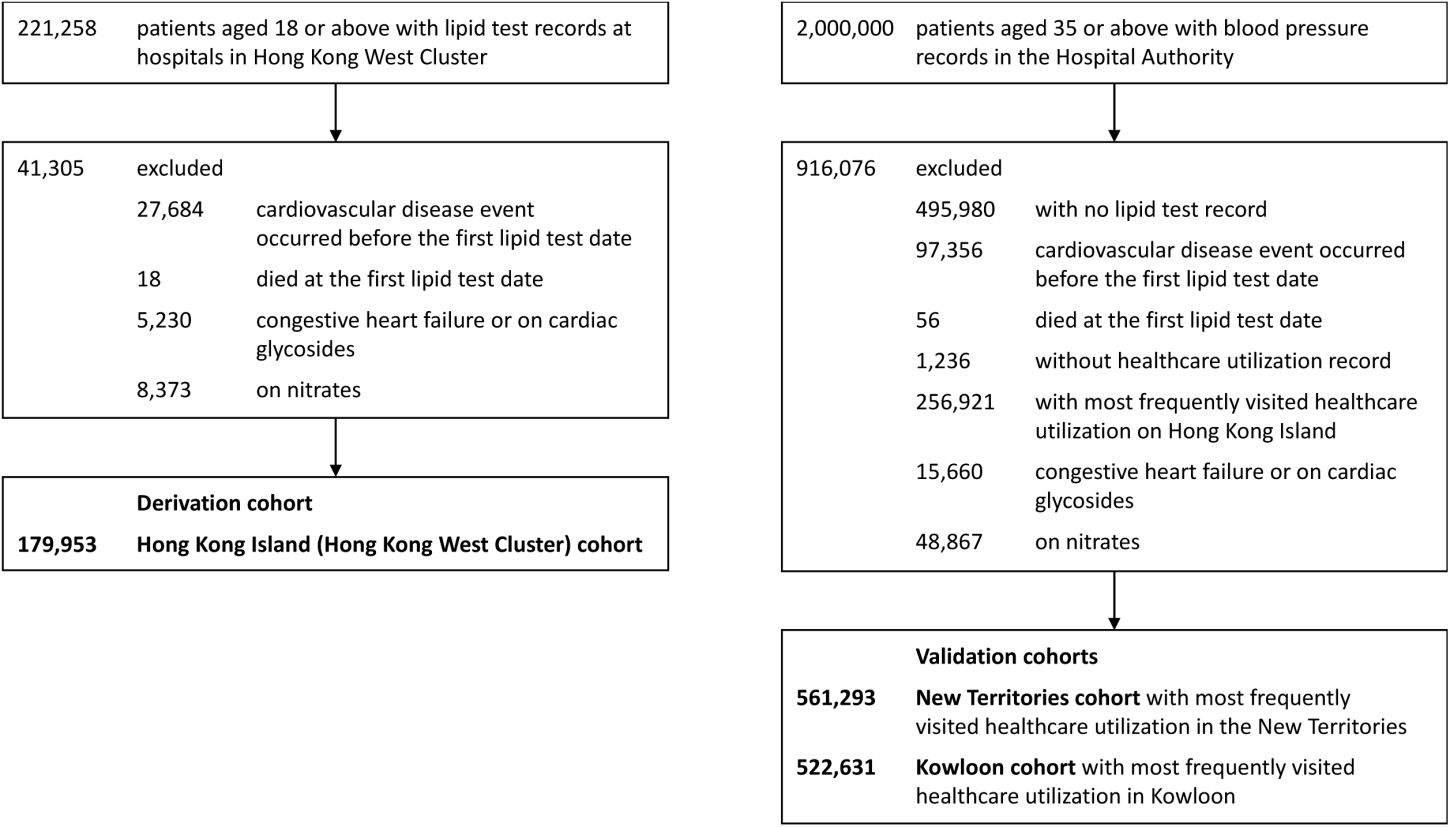
Selection of patients into the study cohorts. N.B. Hong Kong West Cluster is a part of Hong Kong Island.

The study included patients who had used any of the public healthcare services provided by the Hong Kong Hospital Authority (HA) from 1 January 2004–31 December 2019 (Fig 1 and S1 File). Data were accessed in 31 August 2021 for research purpose. The HA is a statutory body and the largest public healthcare provider in Hong Kong, offering government subsidized primary, secondary, and tertiary care to all Hong Kong residents. It covers more than 70% of all hospitalizations in Hong Kong [24]. Previous studies demonstrated high validity of the data source with a positive predictive value of 85% for myocardial infarction and 91% for stroke [25]. The database has been used for over 200 studies published in peer-reviewed journals, including cardiovascular disease and cardiovascular drug studies [25–28], ensuring the creditability of the data source for research purposes [25,26].

Patients were categorized into three cohorts based on their primary location of healthcare utilization in Hong Kong which were Hong Kong Island (Hong Kong West Cluster, HKWC), Kowloon, and New Territories, reflecting the areas where they predominantly received medical care during the study period. The Hong Kong Island (HKWC) cohort was used for model derivation, while the Kowloon and New Territories cohorts were used for model validation. The cohort entry date was the date of their first lipid test in any inpatient or outpatient setting since 2004. Patients were censored (end of follow-up period) at the earliest date of the first record of CVD diagnosis, date of registered death, or study end date (31 December 2019). Patients were excluded from the cohort if they experienced a CVD event before the first lipid test date, or passed away on the same day as the first lipid test.

Since this is a retrospective cohort study, we analyze existing data from past records without directly interacting with or collecting new data from participants. The need for obtaining individual consent to participate in our study was not required by the Institutional Review Board of The University of Hong Kong/Hospital Authority Hong Kong West Cluster. The ethics approval number for this study is UW20–073.

### b. Outcomes and risk variables

The outcome was the first diagnosis of CVD, defined by International Classification of Diseases, Ninth Revision, Clinical Modification (ICD-9-CM) codes. The outcome encompassed a combination of coronary heart disease, ischemic or hemorrhagic stroke, peripheral artery disease, and revascularization (S1 Table).

All available longitudinal data were collected, including information from clinical laboratory tests, disease and medication history, family history of disease, demographic factors, and healthcare utilization (S2 Table). Diagnoses and medical procedures were defined by ICD-9-CM codes (S3 Table), and medication exposure was defined by the British National Formulary (BNF) sections (S4 and S5 Tables).

### c. Model derivation

**Fig 2:**
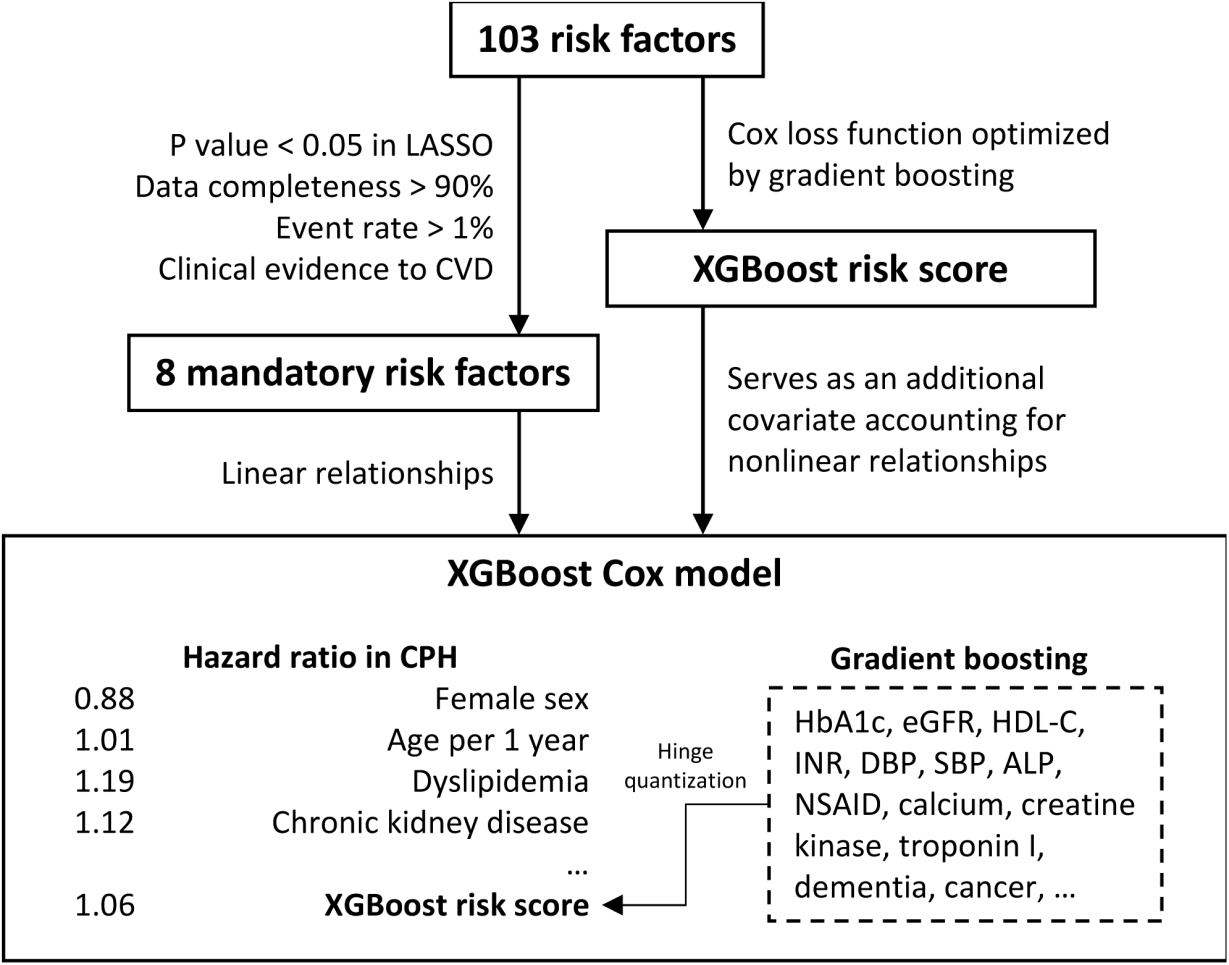
Algorithm design of XGBoost Cox model used in 1°P-CARDIAC full model.

We used the XGBoost Cox model for model derivation, a novel hybrid statistical machine-learning model that we designed (Fig 2). We used a similar approach to develop the P-CARDIAC secondary model which is to predict recurrent CVD risk among people with established CVD risk [29]. The detailed method of model derivation had been described in the previously [29] (S2 and S3 Files). A full model with all available risk variables was built as a Cox proportional hazards (CPH) model with ridge regularization regressed on the mandatory risk variables and the XGBoost risk score [30,31]. Hyperparameters used in XGBoost is optimized by grid search. Ridge regularization is widely used as a stabilizer of regression coefficients, which provides reliable estimates of the hazard ratios of the risk variables. The statistical section of the model is to help identify a shorter list of mandatory risk variables based on clinical evidence, statistical correlation, and data completeness, aiming to strike a balance between model stability and interpretability and clinical relevance to ensure the final set of risk variables were comprehensive and relevant for CVD prognosis. Some risk variables with high relevance to CVD prognosis which do not meet the criteria as mandatory risk variables were also included in the mandatory fields to help improve the prediction. We also built a basic model with the integration of statistically significant and important subset of risk variables for practicality when limited time or information was available.

Multivariate imputation with chained equations (MICE) [32] was used to replace the missing values in clinical laboratory tests. For better statistical reliability and clinical utility, risk variables with a missing rate below 10% (e.g., clinical laboratory tests) and an event rate above 1% (e.g., disease and medication history) were selected. We employed least absolute shrinkage and selection operator (LASSO)^31^ regression to shortlist statistically signiﬁcant (p value<0.05) risk variables.

### d. Model validation

We compared the model performance of 1°P-CARDIAC, PCE, PREDICT, China-PAR, and Framingham (Asian) using the validation cohorts with 1000 bootstrap replicates. The internal consistency of 1°P-CARDIAC model performance was evaluated on the derivation cohort by 100 repeats of 10-fold cross-validation.

Calibration performance was assessed graphically by categorizing patients into deciles of predicted 10-year CVD risk and plotting mean 10-year predicted risk against observed 10-year risk obtained by the Kaplan-Meier method [33]. Recalibration [34] was performed if it improved the calibration curve. Mean and confidence intervals of Harrell’s C statistic [35], calibration-in-the-large [36], and calibration slope were calculated. The calibration slope was the slope of linear regression of the observed risk against the predicted risk of each decile. Brier score was calculated to assess the overall calibration of predictions [37]. The clinical value of the tools was assessed by decision curve analysis [38–40]. Feature importance in XGBoost was assessed by SHAP (SHapley Additive exPlanations) [41].

All analyses were conducted using Python (version 3.9.1), with add-on packages lifelines [42]. This study report is in accordance with the TRIPOD statement [43].

## 3. Results

### a. Study cohorts

For the HKWC derivation cohort, a total of 221,258 patients aged 18 or above with lipid test records were identified. Since lipid profile is a strong predictor of future CVD risk based on current knowledge, individuals with at least one lipid test record were included to ensure comprehensive data for this crucial variable. Subsequently, 27,702 patients were excluded from the cohort whose first CVD event occurred before their first lipid test date or who had died on or before the first lipid test date. Additionally, 13,603 patients with congestive heart failure or those using nitrates or cardiac glycosides were excluded. Overall, 179,953 patients were included in the Hong Kong Island (Hong Kong West Cluster) cohort for model derivation.

For the validation cohorts, a random cohort of 2,000,000 patients aged 35 or above with blood pressure records in HA database was identified. As blood pressure is also an important risk factor for CVD, we included patients with at least one blood pressure record for validation cohort. We excluded 495,980 patients who had no lipid test record during the study period. We excluded 97,412 patients whose first cardiovascular disease event occurred before their first lipid test date or who had died on or before the first lipid test date. Furthermore, we excluded 1,236 patients without healthcare utilization records. To prevent overlap with the derivation cohort, 256,921 patients with the most frequent healthcare utilization on Hong Kong Island were excluded. We further excluded 64,527 patients who had congestive heart failure or were on nitrates or cardiac glycosides. Overall, 561,293 patients were included in the New Territories cohort, and 522,631 patients were included in the Kowloon cohort.

### b. Incidence rates of CVD and baseline characteristics

Table 1 shows the rates of CVD events observed in the three cohorts. The incidence rate per 1,000 person-years was 13–18, with median estimated 10-year event rate falling between 11.7 and 16.2%. During a median follow-up of 6.8 to 7.8 years, 9–14% of patients experienced their first cardiovascular disease event. Regarding the composition of incident CVD events, coronary heart disease (CHD) was the most common outcome, accounting for 55% to 64% of cases, with myocardial infarction representing 10% to 18% of these cases. Stroke followed the second most common outcome, with a proportion of 32% to 43%. The ratio of peripheral artery disease (PAD) was 3–4%.

**Table 1:** Patient characteristics.

	Hong Kong Island (Hong Kong West Cluster)		Kowloon		New Territories	
**Participants**	179,953		522,631		561,293	
**Incident cardiovascular events**	16,965	(9%)	73,551	(14%)	67,189	(12%)
Coronary heart disease	10,877	(64%)	40,783	(55%)	37,562	(56%)
Myocardial infarction	1,710	(10%)	12,921	(18%)	10,566	(16%)
Stroke	5,384	(32%)	31,659	(43a%)	28,450	(42%)
Peripheral artery disease	715	(4%)	2,004	(3%)	1,836	(3%)
Revascularization	2,628	(15%)	3,189	(4%)	3,814	(6%)
*Fatal events	978	(6%)	6,576	(9%)	4,938	(7%)
**Total person-years observed**	1,288,080		4,038,513		4,303,907	
**Event rate per 1000 person-years**	13		18		16	
**Follow-up (years)	6.8	(0.4-15.2)	7.8	(1.0 −15.4)	7.8	(1.2-15.3)
***10-year event rate (%)	11.7	(11.5-11.9)	16.2	(16.1-16.3)	14.1	(13.9-14.2)

*Note: All data in n (%) or median (interquartile range) unless indicated otherwise. All subtypes of incidence events in the Kowloon and New Territories cohorts were significantly different (p value<0.05) compared to the Hong Kong Island (Hong Kong West Cluster) under Chi-square test. Event rate was the incident event divided by total person-years of each cohort. *Deaths within 28 days after incident cardiovascular event. **Median (5th/95th percentile). ***Mean (95% confidence interval), estimated by Kaplan-Meier method.

Table 2 and S6 Table show the baseline characteristics of the risk variables across the three cohorts. The median age at the cohort entry date ranged from 57 to 60 years, and 54–56% of the patients were female. A notable number of patients had comorbidities: 5–7% of patients had dyslipidemia, 39–65% of patients had hypertension, and 20–25% of patients had DM.

**Table 2:** Summary of baseline characteristics for the mandatory risk predictors.

	Hong Kong Island (Hong Kong West Cluster)		Kowloon		New Territories	
**Demographics**						
Age (years)	57	(47-67)	60	(51-70)	58	(50-66)
Female	97,516	(54%)	291,035	(56%)	307,740	(55%)
Male	82,437	(46%)	231,596	(44%)	253,553	(45%)
**Clinical laboratory test**						
HDL cholesterol (mmol/L)	1.3	(1.1-1.6, 1%)	1.3	(1.1-1.6, 1%)	1.4	(1.1-1.6, 1%)
**Disease history**						
Dyslipidemia	13,094	(7%)	29,028	(6%)	28,650	(5%)
Atrial fibrillation	2,029	(1%)	3,393	(1%)	3,365	(1%)
Hypertension	70,636	(39%)	337,414	(65%)	354,078	(63%)
Diabetes	35,469	(20%)	128,156	(25%)	135,771	(24%)
Chronic kidney disease	1,290	(1%)	1,712	(0%)	1,325	(0%)

*Note: HDL cholesterol = high-density lipoprotein cholesterol. All data in n (%), or median (interquartile range), or median (interquartile range, proportion of missing data) unless indicated otherwise. All risk variables in the Kowloon and New Territories cohorts were significantly different (p value<0.05) compared to the Hong Kong Island (Hong Kong West Cluster) under Chi-square test (categorical risk variables) or in T-test (numerical risk variables).

### c. Model derivation

We identified 103 risk variables and eight of them are mandatory risk variables (Table 3 and S2 Table) due to their statistically significant and clinically relevance in CVD prognosis. The mandatory risk variables were age, sex, high-density lipoprotein cholesterol, disease history of dyslipidemia, diabetes, atrial fibrillation, hypertension, and chronic kidney disease. We also included another 8 clinically important variables (including low-density lipoprotein, HbA1c, diastolic and systolic blood pressure, prior use lipid-modifying drug, antihypertensive, antidiabetic and antiplatelet use) in the mandatory fields to improve the reliability of risk prediction. For laboratory measures in the mandatory fields, imputation was performed using the population means of the derivation cohort to generate the prediction score. The rest of the risk variables were considered supplementary, offering the potential to improve prediction accuracy if available. The 1°P-CARDIAC basic model was trained on mandatory risk variables to capture their linear effects, while the full model was trained in addition to a XGBoost risk score representing the complex interaction effects of all 103 risk variables. The performance of 1°P-CARDIAC on the derivation cohort had good performance as the validation with a C statistic of 0.87 and 0.75 for the full and basic models, respectively (S3 Fig and S12 Table). SHAP (SHapley Additive exPlanations) analysis revealed that novel biomarkers such as creatine kinase, INR, and eGFR—alongside traditional risk factors—contributed meaningfully to the XGBoost component’s predictions. These variables captured nonlinear associations (e.g., U-shaped relationships for electrolyte levels) and interaction effects, enhancing the hybrid model’s predictive accuracy while retaining clinical plausibility in CVD pathophysiology (S4 Fig).

**Table 3:** Adjusted hazard ratios in 1°P-CARDIAC models.

	Basic model (Mandatory risk variables)		Full model (Mandatory + Supplementary risk variables)	
	HR (95% CI)	p value	HR (95% CI)	p value
**Demographics**				
Age (years)	1.04 (1.04-1.05)	<0.05	1.01 (1.00-1.01)	<0.05
Female	0.62 (0.60-0.64)	<0.05	0.88 (0.86-0.91)	<0.05
**Clinical laboratory test**				
HDL cholesterol per 1 mmol/L	0.70 (0.67-0.73)	<0.05	0.87 (0.84-0.91)	<0.05
**Disease history**				
Dyslipidemia	1.89 (1.81-1.97)	<0.05	1.19 (1.14-1.24)	<0.05
Atrial fibrillation	1.60 (1.45-1.77)	<0.05	1.02 (0.92-1.12)	0.71
Hypertension	1.19 (1.16-1.23)	<0.05	1.03 (1.00-1.06)	0.38
Diabetes	1.29 (1.25-1.33)	<0.05	1.01 (0.98-1.05)	0.06
Chronic kidney disease	2.04 (1.81-2.29)	<0.05	1.12 (1.00-1.25)	0.06
**XGBoost risk score**			1.06 (1.06-1.06)	<0.05

*Note: Abbreviations: HDL cholesterol = high-density lipoprotein cholesterol. HR = hazard ratio, CI = confidence interval.

### d. Model validation

**Fig 3:**
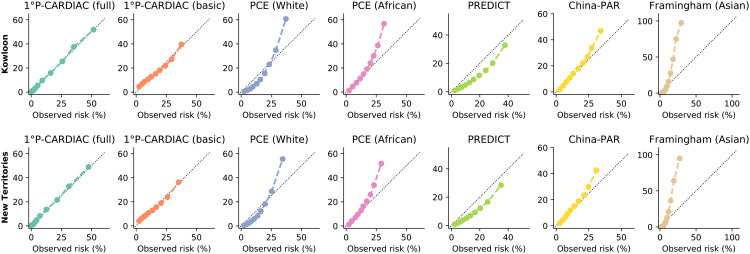
Calibration plots on validation cohorts.

**Fig 4:**
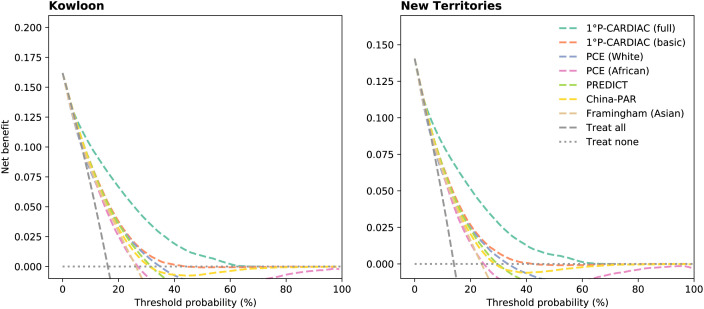
Decision curve analysis on validation cohorts.

Validation results for the two cohorts were similar. The validation showed that the 1°P-CARDIAC full model was superior to the other tools, exhibiting a clear competitive edge in discrimination, calibration, and clinical values. The full model had the highest C-statistic of 0.82 across the two cohorts, indicating its capability to differentiate subtle variations in patient risks effectively (Table 4). The plots comparing predicted versus observed risks displayed high precision across all deciles, with calibration slope and calibration-in-the-large values closely approximating the ideal targets of 1 and 0, respectively (Fig 3 and S7 and S8 Tables), demonstrating the reliability of its prediction for clinical applications. Regarding decision curve analysis, the full model had the highest net benefit across the largest range of threshold risks (Fig 4), highlighting its potential in clinical decision making.

**Table 4:** Mean (95% CI) of Harrell’s C statistic on validation cohorts.

	Kowloon	New Territories
1°P-CARDIAC (full)	0.82 (0.82, 0.82)	0.82 (0.82, 0.82)
1°P-CARDIAC (basic)	0.71 (0.71, 0.71)	0.72 (0.72, 0.72)
PCE (White)	0.70 (0.70, 0.70)	0.71 (0.71, 0.71)
PCE (African)	0.68 (0.68, 0.68)	0.68 (0.68, 0.68)
PREDICT	0.71 (0.71, 0.71)	0.72 (0.72, 0.72)
China-PAR	0.69 (0.69, 0.69)	0.70 (0.70, 0.70)
Framingham (Asian)	0.69 (0.69, 0.69)	0.70 (0.70, 0.70)

*Note: CI = confidence interval. Values were measured from 1000 bootstrap replicates. Results of P-CARDIAC (basic), PCE (African), PREDICT, and China-PAR were after recalibration.

The basic model yielded the second-best validation results, with a C statistic of 0.71 and 0.72 for the two cohorts. It exhibited good performance in calibration and decision curve analysis and was inferior only to the full model. The validations of PCE, PREDICT, China-PAR, and Framingham (Asian) were underperformed with C statistics ranging between 0.68 and 0.72 for both validation cohorts. As a Chinese-specific model, China-PAR had good calibration for low-risk patients but overestimated the risk for high-risk patients. In addition, the Brier score of P-CARDIAC also achieved the best overall calibration among the tools being compared (S13 Table).

Recalibration provided improvements for 1°P-CARDIAC (basic), PCE (African), PREDICT, and China-PAR. The results before recalibration are shown in S1 and S2 Figs and S9–S11 Tables.

### e. Website design

The website interface at p-cardiac.com was designed to be flexible and interactive, complemented with our secondary model (see S4 File for sample screenshots). Users can input the mandatory risk variables for a quick, reliable evaluation of CVD risk. They can further input the supplementary risk variables for a more comprehensive and accurate evaluation.

## 4. Discussion

To our knowledge, 1°P-CARDIAC is the first CVD risk prediction model for primary prevention using a novel hybrid statistical machine-learning model that accounts for a wide array of parameters in a contemporary Chinese population. It was validated on the largest contemporary Chinese cohort to date and demonstrated superior performance compared to existing CVD risk scores, such as PCE, PREDICT, and China-PAR. The 1°P-CARDIAC full model showed clear improvement over the basic model, underscoring the potential of our novel method, which can effectively leverage the power of big data. Overall, 1°P-CARDIAC represents a significant advance in CVD risk prediction for Chinese.

Our findings emphasize the importance of incorporating changes in treatment and other socio-economic patterns when developing CVD risk-prediction models. With the widespread availability of ERH and the emergence of new lipid-modifying drugs like PCSK9 inhibitors and inclisiran, our model, developed with EHR, could be easily recalibrated with up-to-date data. In China, the percentage of patients taking lipid-modifying drugs increased from 3.4–3.5% in 2000 [44] to 7–10% in our cohort in 2004 with longitudinal data up to 2021. In comparison to China-PAR, a risk assessment tool for 10-year ASCVD developed for the Chinese population, 1°P-CARDIAC demonstrated superior performance [9]. This difference could potentially explain by China-PAR less up-to-date derivation cohort which was established before 2000 and followed-up till 2015. Another potential reason was the differences in lifestyle and patient characteristics between our Hong Kong cohorts and the cohorts used in China-PAR, particularly in the more northern regions of China. Hong Kong is a city in southeast China that has been urbanized for a longer time with long history of Western cultural influence since the 1800s, compared to mainland China where the drastic change in lifestyle and culture only started since the economic reform in 1980s. While China-PAR performed well in predicting risk for low-risk patients, it tended to overestimate risk for high-risk patients in our contemporary cohorts. This highlights the importance of considering changes in treatment other than lipid-modifying drugs to avoid overestimating risk in the current population [21,45,46]. Our study suggested that incorporating multiple types of prior treatments as risk variables in the model can effectively account for the effect of treatment in CVD primary prevention.

Evidence suggests that simply recalibrating a universal risk score to match a specific ethnicity may not be sufficient for accurate risk prediction [21]. The validation of PREDICT in our study supported that consideration of multiple ethnicities as a risk variable may still be inadequate in accurate CVD risk prediction. Furthermore, when comparing risk assessment tools developed with Chinese population cohort, 1°P-CARDIAC showed better performance over China-PAR when machine-learning technique was used considering a much wider array of parameters. This highlights the importance of leveraging big data to improve public health outcomes. Improving risk prediction accuracy across diverse populations requires to collect and consider more variables and adopt properly tailored methods which can identify unique patterns and factors that are relevant to each specific population. Our novel hybrid statistical machine-learning model demonstrated the feasibility of considering a wide array of parameters without compromising the rigor of the analysis.

The Framingham (Asian) underperformed among the validation cohorts in our study. The disparity in performance can be attributed to the differences in sampling of the study cohort. The Framingham (Asian) was recalibrated using cohorts consisting of generally healthy middle-aged participants [23], whereas our study population consisted of individuals who require medical attention, including those sought medical treatment in HA clinicals and hospitals for various purposes, not necessarily CVD related. The ultimate goal of 1°P-CARDIAC is to be implemented in the healthcare system, where EHR is readily available to be fitted for risk estimation. It will potentially facilitate healthcare providers in providing CVD primary prevention and to improve referral between primary and specialty care. The setting where we anticipate 1°P-CARDIAC could be most useful is different from that of Framingham (Asian).

Our innovative model also demonstrated both efficacy and versatility in harnessing the power of big data. In our P-CARDIAC secondary prevention model for people with established CVD risk, the hybrid approach had already been utilized with superior performance compared to traditional methods [29]. The validity and good performance of 1°P-CARDIAC described in this paper further illustrates the power of our novel method for CVD risk prediction and prevention. The P-CARDIAC and 1°P-CARDIAC together will provide comprehensive risk prediction for people at risk or already experienced CVD risk which could serve as an objective measure for allocating relevant medical resources based on different risk level. These models also highlight the potential for our approach to serve as a common method for predictive modelling in various medical domains beyond the scope of CVD-related areas. In summary, our approach possesses a potential and broad impact on medicine by effectively utilizing the readily available EHR in risk assessment.

This study has limitations. First, while the basic model includes a concise set of risk variables for easy input, additional factors such as inflammatory biomarkers (c-reactive protein and neutrophils) [47], smoking and drinking habit were only considered in the full model as supplementary risk variables. They were not included in the basic model due to high missingness (S6 Table) in our derivation cohort which was a common limitation in many EHR. However, we had good internal validation with considerations of these readings in our validation cohorts, reassuring these risk variables, if available, would support a better CVD risk prediction. Second, 1°P-CARDIAC was developed using real-world data, so any changes in clinical practices in the future may impact the predictive accuracy. Diverse demographical population groups from China either in rural or urban would also change the estimated results. We believed the recalibration by different subpopulation in China would robust our model. Further study should be conducted to enhance the model’s applicability and accuracy across diverse demographic groups. Third, although time information is available in each patient record, the current version of 1°P-CARDIAC does not consider the time-varying effects of risk variables. However, incorporating this feature could improve the performance of the model in the future development. Lastly, 1°P-CARDIAC serves as a risk stratification tool to better optimize healthcare resources allocation, rather than a diagnostic tool. Therefore, a composite risk score was given for a spectrum of CVD diseases, rather than disease-specific scores. While technological advances enable us to leverage big data, we believe integrating genetic biomarkers associated with CVD, echocardiogram and dietary habits in the future can offer more precise estimation. We hope that the empathy of healthcare providers and their connection with their patients, which influences the best healthcare decisions, cannot be replaced by artificial intelligence in the near future.

## 5. Conclusions

We developed and validated 1°P-CARDIAC, a CVD risk prediction model for primary prevention applying a novel hybrid statistical and machine-learning approach. The model accommodates the complex treatment of CVD primary prevention and other socio-economic patterns in a contemporary Chinese population, resulting in promising performance compared to other existing tools. Though the utilization of novel hybrid statistical machine-learning approach, 1°P-CARDIAC highlighted both effectiveness and versatility in harnessing the power of big data and which is able to serve as a common method for primary CVD prevention to improve public health. This study also demonstrated the capability of utilizing real-world data to provide guidance for early intervention, encouraging lifestyle modification and medication compliance to prevent the occurrence of CVD events, thus reducing the related healthcare burden.

## Supporting information

S1 File Details of the data source. (DOCX)

S2 File Gradient boosting Cox proportional hazards modeling using XGBoost. (DOCX)

S3 File Design of the hinge loss-like function. (DOCX)

S4 File Screenshots and clinical example of the 1°P-CARDIAC website interface. (DOCX)

S1 Table Definition of cardiovascular disease. (DOCX)

S2 Table Summary of included variables. (DOCX)

S3 Table Disease list. (DOCX)

S4 Table Drug list. (DOCX)

S5 Table Lipid-modifying drugs subclasses. (DOCX)

S6 Table Summary of supplementary variables. (DOCX)

S7 Table Mean (95% CI) of calibration slope on validation cohorts. (DOCX)

S8 Table Mean (95% CI) of calibration-in-the-large on validation cohorts. (DOCX)

S9 Table Mean (95% CI) of Harrell’s C statistic on validation cohorts before recalibration. (DOCX)

S10 Table Mean (95% CI) of calibration slope on validation cohorts before recalibration. (DOCX)

S11 Table Mean (95% CI) of calibration-in-the-large on validation cohorts before recalibration. (DOCX)

S12 Table Discrimination and calibration performance of 1°P-CARDIAC on derivation cohort. (DOCX)

S13 Table Brier score on validation cohorts. (DOCX)

S1 Fig Calibration plots of 1°P-CARDIAC (basic), PCE (African), PREDICT, and China-PAR before recalibration. (DOCX)

S2 Fig Decision curves of 1°P-CARDIAC (basic), PCE (African), PREDICT, and China-PAR before recalibration. (DOCX)

S3 Fig Calibration plots for the 1°P-CARDIAC (full) model in the Hong Kong Island (Hong Kong West Cluster) derivation cohort with 95% Confidence interval. (DOCX)

S4 Fig SHAP (SHapley Additive exPlanations) summary plot of the P-CARDIAC hybrid model. (DOCX)
