# Supplementary material for: Primary prevention cardiovascular disease risk prediction model for contemporary Chinese (1°P-CARDIAC): Model derivation and validation using a hybrid statistical and machine-learning approach
Source: PLoS One. 2025 Jul 28;20(7):e0322419. doi: 10.1371/journal.pone.0322419 (PMC12303301; doi:10.1371/journal.pone.0322419)
Supplement: S1 File — (DOCX) [file pone.0322419.s001.docx]

**Supplementary Information 1. Details of the data source**

The Hong Kong Island (Hong Kong West Cluster) cohort, identified by the Hospital Authority, included all patients aged 18 or above at the time they received their lipid test at a hospital located in the Hong Kong West Cluster between 1 January 2004 and 31 December 2019. 1°P-CARDIAC was derived from the Hong Kong Island (Hong Kong West Cluster) cohort.

For the Kowloon and New Territories cohorts, a two million patient cohort was retrieved from the Hospital Authority database, which included all patients aged 35 or above at the time they had their blood pressure recorded in the Hospital Authority between 1 January 2005 and 31 December 2019. External validation was completed using the Kowloon and New Territories cohorts to ensure no overlap with the model-derived cohort.
