# Supplementary material for: Primary prevention cardiovascular disease risk prediction model for contemporary Chinese (1°P-CARDIAC): Model derivation and validation using a hybrid statistical and machine-learning approach
Source: PLoS One. 2025 Jul 28;20(7):e0322419. doi: 10.1371/journal.pone.0322419 (PMC12303301; doi:10.1371/journal.pone.0322419)
Supplement: S2 File — (DOCX) [file pone.0322419.s002.docx]

**Supplementary Information 2. Gradient boosting Cox proportional hazards modeling using XGBoost**

The Cox model is expressed by the hazard function, denoted by h(t). Briefly, the hazard function can be interpreted as the risk of dying (or other event of interest happening) at time t.

ln(h(t)) = ln(h0(t)) + <w,x>

Where:

x is a vector in Rd representing the features.

w is a vector consisting of d coefficients, each corresponding to a feature.

⟨⋅,⋅⟩ is the usual dot product in Rd.

ln(⋅) is the natural logarithm.

the term h0(t) is the baseline hazard.

XGBoost revises the model as follows to make Cox work with gradient boosting:

ln(h(t)) = ln(h0(t)) + T(x)

where T(x) represents the output from a decision tree ensemble, given input x. The goal for XGBoost is to maximize the (log) likelihood by fitting a good tree ensemble T(x).
