# Supplementary material for: Primary prevention cardiovascular disease risk prediction model for contemporary Chinese (1°P-CARDIAC): Model derivation and validation using a hybrid statistical and machine-learning approach
Source: PLoS One. 2025 Jul 28;20(7):e0322419. doi: 10.1371/journal.pone.0322419 (PMC12303301; doi:10.1371/journal.pone.0322419)
Supplement: S3 File — (DOCX) [file pone.0322419.s003.docx]

**Supplementary Information 3. Design of the hinge loss-like function**

f(p) = max(0, p-t)

t is the threshold with a value of 20.

p is the discrete percentile of the hazard ratio for all involved patients.
