# Supplementary material for: Primary prevention cardiovascular disease risk prediction model for contemporary Chinese (1°P-CARDIAC): Model derivation and validation using a hybrid statistical and machine-learning approach
Source: PLoS One. 2025 Jul 28;20(7):e0322419. doi: 10.1371/journal.pone.0322419 (PMC12303301; doi:10.1371/journal.pone.0322419)

**Supplementary Information 4. Screenshots and clinical example of the 1°P-CARDIAC website interface**

1. Fill in the mandatory fields


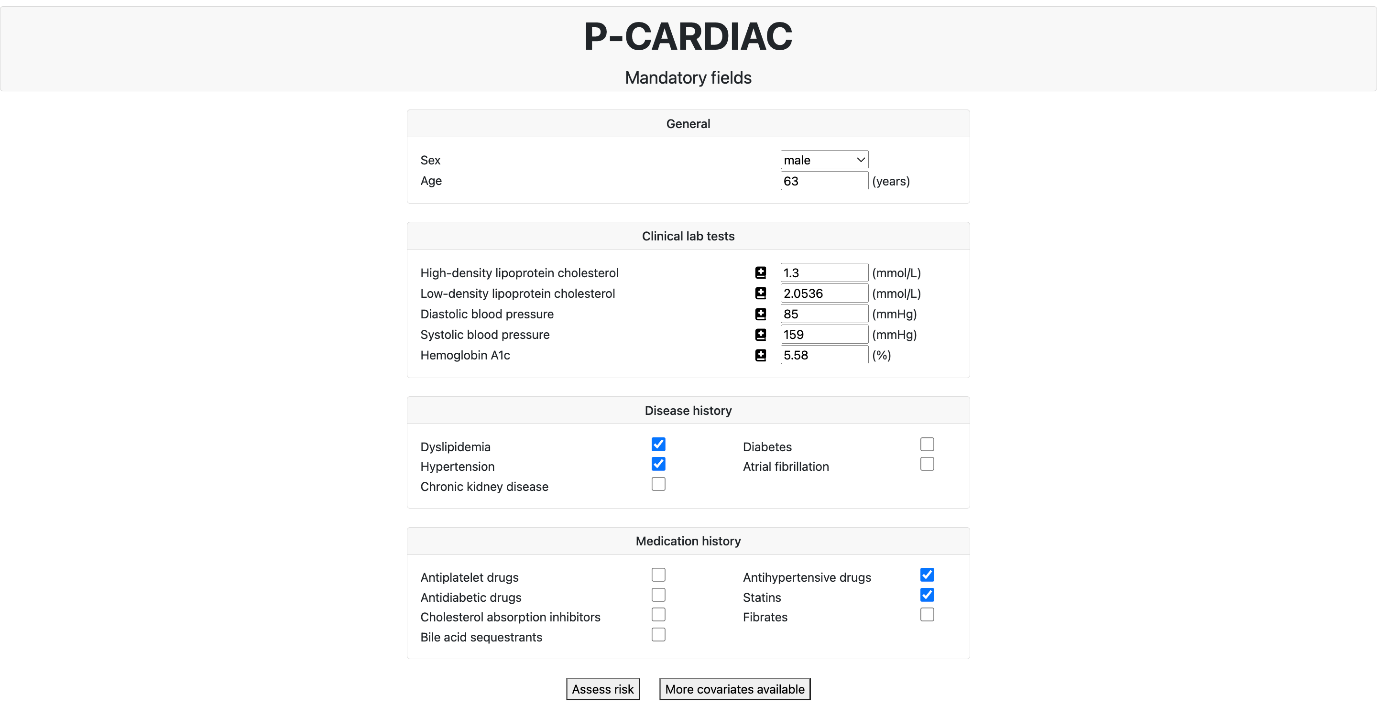


2. Assess the risk based on only mandatory risk variables


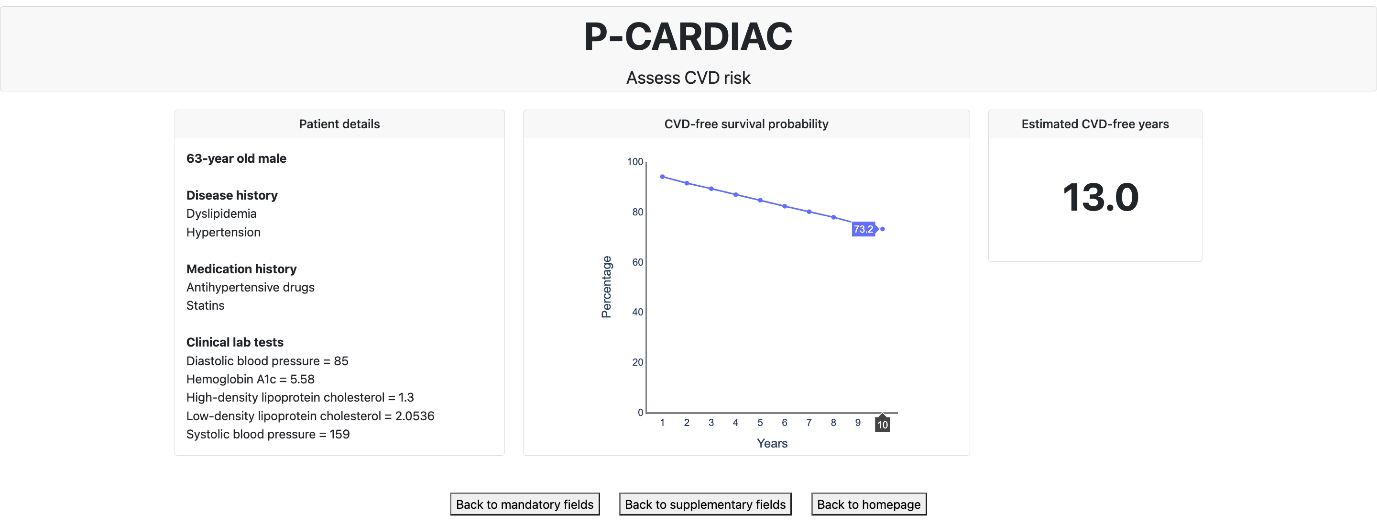


3. Fill in the supplementary fields


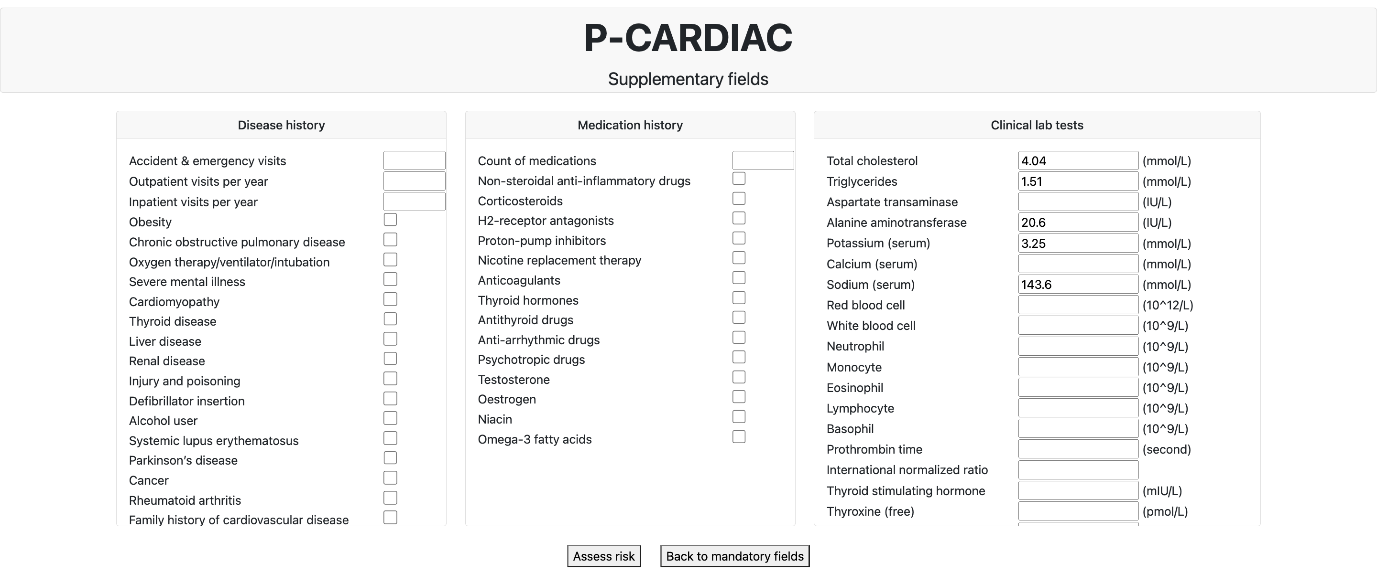


4. Assess risk based on both mandatory and supplementary risk variables


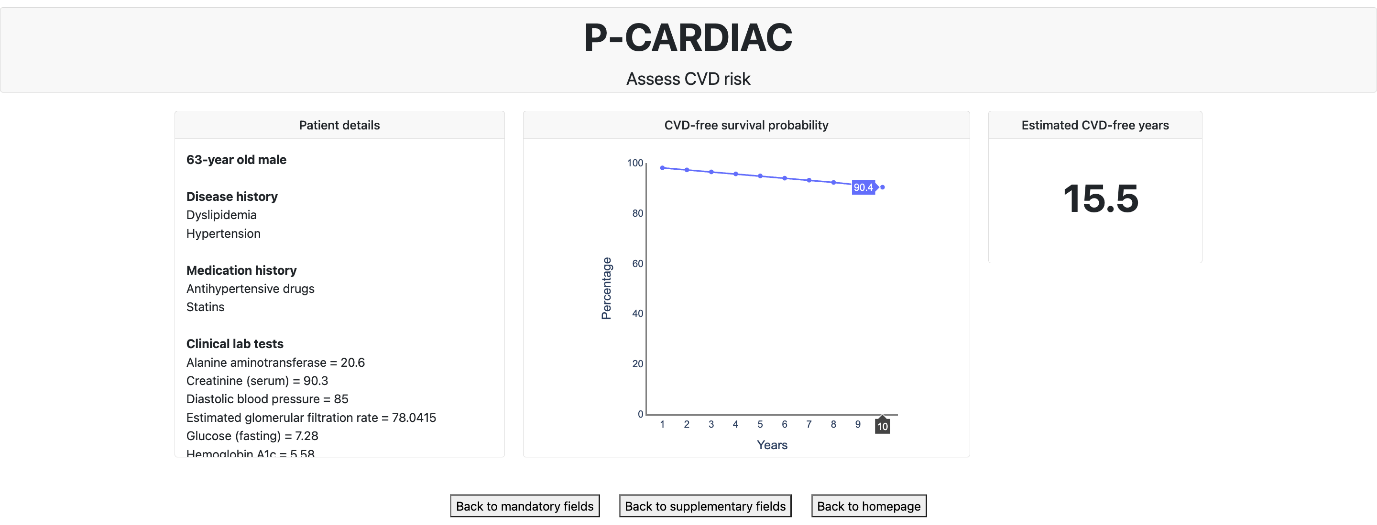

Supplement: S4 File — (DOCX) [file pone.0322419.s004.docx]
