# Supplementary material for: Primary prevention cardiovascular disease risk prediction model for contemporary Chinese (1°P-CARDIAC): Model derivation and validation using a hybrid statistical and machine-learning approach
Source: PLoS One. 2025 Jul 28;20(7):e0322419. doi: 10.1371/journal.pone.0322419 (PMC12303301; doi:10.1371/journal.pone.0322419)
Supplement: S1 Table — (DOCX) [file pone.0322419.s005.docx]

**Supplementary Table 1. Definition of cardiovascular disease**

| **Diagnosis** | **ICD-9** |
| --- | --- |
| Peripheral artery disease | 440, 443.9 |
| Coronary heart disease | 410-414, 429.2, V45.81 |
| Myocardial infarction | 410 |
| Stroke | 430, 431, 432, 433.01, 433.11, 433.21, 433.31, 433.81, 433.91, 434, 435, 436, 437.0, 437.1 |
| **Procedure** |  |
| Revascularization | 36.01-36.20 |
