# Supplementary material for: Primary prevention cardiovascular disease risk prediction model for contemporary Chinese (1°P-CARDIAC): Model derivation and validation using a hybrid statistical and machine-learning approach
Source: PLoS One. 2025 Jul 28;20(7):e0322419. doi: 10.1371/journal.pone.0322419 (PMC12303301; doi:10.1371/journal.pone.0322419)
Supplement: S2 Table — (DOCX) [file pone.0322419.s006.docx]

**Supplementary Table 2. Summary of included variables**

| Categories (number of covariates) | Risk variables |
| --- | --- |
| **Demographic factors (2)** | age*, sex* |
| **Family history of disease (2)** | diabetes, cardiovascular disease |
| **Healthcare utilization (3)** | accident and emergency visits per year, inpatient visits per year, outpatient visits per year |
| **Clinical laboratory tests (38)** | high-density lipoprotein cholesterol*, low-density lipoprotein cholesterol*, hemoglobin A1c*, diastolic blood pressure*, systolic blood pressure*, aspartate transaminase, alanine aminotransferase, neutrophil, creatine kinase (total), prothrombin time, potassium (serum), estimated glomerular filtration rate, triglycerides, basophil, arterial partial pressure of oxygen, albumin, international normalized ratio, bicarbonate (serum), glucose (fasting), erythrocyte sedimentation rate, free thyroxine, troponin I, bilirubin (total), C-reactive protein, total cholesterol, blood pH, thyroid stimulating hormone, lymphocyte, creatinine (serum), platelet, red blood cell, calcium (serum), white blood cell, alkaline phosphatase, sodium (serum), eosinophil, hemoglobin, monocyte |
| **Medication history (22)** | antiplatelet drugs*, statins*, antihypertensive drugs*, antidiabetic drugs*, non-steroidal anti-inflammatory drugs, corticosteroids, proton-pump inhibitors, H2-receptor antagonists, anticoagulants, nicotine replacement therapy, antiarrhythmic drugs, antithyroid drugs, oestrogen, psychotropic drugs, thyroid hormones, testosterone, fibrates*, niacin, cholesterol absorption inhibitors*, bile acid sequestrants*, omega-3 fatty acids, count of medications |
| **Disease history (36)** | dyslipidemia*, diabetes*, atrial fibrillation*, hypertension*, chronic kidney disease*, thyroid disease, arrhythmia and conduction disorders, obesity, hypothyroidism, cardiac wall/valve/shunt replacement/repairment, oxygen therapy/ventilator/intubation, asthma, injury and poisoning, alcohol user, cardiomyopathy, Parkinson’s disease, defibrillator insertion, major organ bleeding, severe mental illness, dementia, pacemaker implantation, liver disease, chronic obstructive pulmonary disease, cancer, rheumatoid arthritis, renal disease, smoker, muscle pain or myopathy or rhabdomyolysis, dialysis, Creutzfeldt-Jakob disease, cardioversion, nephrotic syndrome, systemic lupus erythematosus, migraine, Down’s syndrome, erectile dysfunction |

*Risk variables included in the mandatory fields. H2 = histamine type 2.
