# Supplementary material for: Primary prevention cardiovascular disease risk prediction model for contemporary Chinese (1°P-CARDIAC): Model derivation and validation using a hybrid statistical and machine-learning approach
Source: PLoS One. 2025 Jul 28;20(7):e0322419. doi: 10.1371/journal.pone.0322419 (PMC12303301; doi:10.1371/journal.pone.0322419)
Supplement: S3 Table — (DOCX) [file pone.0322419.s007.docx]

**Supplementary Table 3. Disease list.**

| **Disease/Symptoms** | **ICD-9-CM code** |
| --- | --- |
| Atrial fibrillation | 427.3 |
| Renal disease | 403.01, 403.11, 403.91, 404.02, 404.03, 404.12, 404.13, 404.92, 404.93, 580, 582, 583.0-583.7, 585-587, 588.0, 589, 590, 593.0-593.2, 593.6, 593.8, 593.9, 599.7, 753.0-753.4, 966.1, V42.0, V45.1, V56 |
| Chronic kidney disease | 585 |
| Dialysis | 585.9, V56.0, V56.8, 39.95 |
| Congestive heart failure | 428 |
| Diabetes | 250 |
| Down’s syndrome | 758.0 |
| Hypertension | 401-405 |
| Arrhythmia and conduction disorders | 426, 427 |
| Cardiomyopathy | 425 |
| Angina | 413 |
| Coronary artery bypass graft | 414.04, V45.81 |
| Myocardial infarction | 410 |
| Dyslipidemia | 272 |
| Thyroid disease | 240-244 |
| Liver disease | 570-573 |
| Migraine | 346 |
| Nephrotic syndrome | 581 |
| Rheumatoid arthritis | 446.5, 710.0-710.4, 714.0-714.3, 725 |
| Several mental illnesses | 290-319 |
| Systemic lupus erythematosus | 710.0 |
| Obesity | 278 |
| Dementia | 290, 291, 292.82, 294, 331 |
| Chronic obstructive pulmonary disease | 490-492, 494, 496 |
| Asthma | 493 |
| Alcohol use | 265.2, 291, 303, 305.0, 357.5, 425.5, 535.3, 571.0- 571.3, 980, V11.3 |
| Smoker | 305.1, V15.82, V15.83, 649.0 |
| Cancer | 140-209, 230-239 |
| Pacemaker implantation | 37.7, 37.8 |
| Defibrillator insertion | 37.94-37.98 |
| Cardioversion | 99.61 |
| Cardiac wall/valve/shunt replacement/repairment | 39.0-39.2 |
| Echocardiography | 37.28 |
| Heart transplantation | 37.51 |
| Oxygen therapy/ventilator/intubation | 00.49, 93.90, 96.01-96.05, 96.7 |
| Erectile dysfunction | 607.84 |
| Major organ bleeding | 578.0, 578.1 |
| Muscle pain, myopathy, or rhabdomyolysis | 728.8, 729.9, 791.3, 781.99 |
| Injury and poisoning | 800-989 |
| Parkinson’s disease | 332 |
| Huntington’s disease | 333.4 |
| Mild cognitive impairment | 331.83 |
| Memory loss | 780.93 |
| Creutzfeldt-Jakob disease | 046.1 |
| Hypothyroidism | 243-244 |
