# Supplementary material for: Primary prevention cardiovascular disease risk prediction model for contemporary Chinese (1°P-CARDIAC): Model derivation and validation using a hybrid statistical and machine-learning approach
Source: PLoS One. 2025 Jul 28;20(7):e0322419. doi: 10.1371/journal.pone.0322419 (PMC12303301; doi:10.1371/journal.pone.0322419)
Supplement: S4 Table — (DOCX) [file pone.0322419.s008.docx]

**Supplementary Table 4. Drug list**

| **Drug class** | **BNF chapter** |
| --- | --- |
| Corticosteroids | 1.5.2, 1.7.2, 3.2, 6.3, 8.2.2,10.1.2, 11.4.1, 13.4 |
| H2-receptor antagonists | 1.3.1 |
| Proton-pump inhibitors | 1.3.5 |
| Anti-arrhythmic drugs | 2.3.2 |
| Psychotropic drugs | 4.1, 4.2, 4.3, 4.4 |
| Antihypertensive drugs | 2.2, 2.4, 2.5.1, 2.5.2, 2.5.4, 2.5.5, 2.6.2 |
| Anticoagulants | 2.8.1, 2.8.2 |
| Antiplatelet drugs | 2.9 |
| Antidiabetic drugs | 6.1.1.1, 6.1.1.2, 6.1.2.1, 6.1.2.2, 6.1.2.3 |
| Lipid-modifying drugs* | 2.12 |
| Nicotine replacement therapy | 4.10.2 |
| Oestrogen | 6.4.1 |
| Testosterone | 6.4.2 |
| Non-steroidal anti-inflammatory drugs | 10.1.1 |
| Thyroid hormones | 6.2.1 |
| Antithyroid drugs | 6.2.2 |

*Further distinguish the subclasses based on drug names. H2 = histamine type 2.
