# Supplementary material for: Primary prevention cardiovascular disease risk prediction model for contemporary Chinese (1°P-CARDIAC): Model derivation and validation using a hybrid statistical and machine-learning approach
Source: PLoS One. 2025 Jul 28;20(7):e0322419. doi: 10.1371/journal.pone.0322419 (PMC12303301; doi:10.1371/journal.pone.0322419)
Supplement: S5 Table — (DOCX) [file pone.0322419.s009.docx]

**Supplementary Table 5. Lipid-modifying drugs subclasses**

| **Subclass** | **Drug name** |
| --- | --- |
| Statins | Atorvastatin, Fluvastatin, Lovastatin, Pravastatin, Rosuvastatin, Simvastatin |
| Fibrates | Bezafibrate, Clofibrate, Fenofibrate, Gemfibrozil |
| Niacin | Nicotinic acid, Nicotinate, Tredaptive, Acipimox |
| PCSK9 inhibitors | Alirocumab, Evolocumab |
| Cholesterol absorption inhibitors | Ezetimibe |
| Bile acid sequestrants | Cholestyramine |
| Omega-3 fatty acids | Maxepa |
| Vytorin | Vytorin |
| Others | Benfluorex, Probucol |

PCSK9 = Proprotein convertase subtilisin/kexin type 9.
