# Supplementary material for: Primary prevention cardiovascular disease risk prediction model for contemporary Chinese (1°P-CARDIAC): Model derivation and validation using a hybrid statistical and machine-learning approach
Source: PLoS One. 2025 Jul 28;20(7):e0322419. doi: 10.1371/journal.pone.0322419 (PMC12303301; doi:10.1371/journal.pone.0322419)
Supplement: S6 Table — (DOCX) [file pone.0322419.s010.docx]

| **Supplementary Table 6. Summary of supplementary variables** | | | | | | |
| --- | --- | --- | --- | --- | --- | --- |
|  | **Hong Kong Island (Hong Kong West Cluster)** | | **Kowloon** | | **New Territories** | |
| **Disease history** |  |  |  |  |  |  |
| Obesity | 775 | (0%) | 855 | (0%) | 971 | (0%) |
| Thyroid disease | 2932 | (2%) | 6138 | (1%) | 5785 | (1%) |
| Arrhythmia and conduction disorders | 4377 | (2%) | 6875 | (1%) | 6678 | (1%) |
| Oxygen therapy/ventilator/intubation | 946 | (1%) | 2517 | (0%) | 2014 | (0%) |
| Nephrotic syndrome | 329 | (0%) | 434 | (0%) | 397 | (0%) |
| Hypothyroidism | 561 | (0%) | 1493 | (0%) | 1622 | (0%) |
| Cardiac wall/valve/shunt replacement/repairment | 108 | (0%) | 41 | (0%) | 44 | (0%) |
| Erectile dysfunction | 1 | (0%) | 4 | (0%) | 7 | (0%) |
| Asthma | 1362 | (1%) | 2649 | (1%) | 2858 | (1%) |
| Injury and poisoning | 10511 | (6%) | 30818 | (6%) | 34490 | (6%) |
| Alcohol user | 976 | (1%) | 1806 | (0%) | 2154 | (0%) |
| Cardiomyopathy | 413 | (0%) | 223 | (0%) | 290 | (0%) |
| Parkinson’s disease | 446 | (0%) | 794 | (0%) | 723 | (0%) |
| Major organ bleeding | 271 | (0%) | 537 | (0%) | 597 | (0%) |
| Severe mental illness | 13109 | (7%) | 27297 | (5%) | 35894 | (6%) |
| Dementia | 1714 | (1%) | 4285 | (1%) | 4341 | (1%) |
| Pacemaker implantation | 540 | (0%) | 456 | (0%) | 524 | (0%) |
| Liver disease | 4202 | (2%) | 5771 | (1%) | 5119 | (1%) |
| Chronic obstructive pulmonary disease | 1740 | (1%) | 5570 | (1%) | 5260 | (1%) |
| Cancer | 11396 | (6%) | 16265 | (3%) | 14601 | (3%) |
| Rheumatoid arthritis | 1937 | (1%) | 1894 | (0%) | 1624 | (0%) |
| Renal disease | 4053 | (2%) | 8376 | (2%) | 9279 | (2%) |
| Muscle pain, myopathy or rhabdomyolysis | 238 | (0%) | 557 | (0%) | 579 | (0%) |
| Dialysis | 1130 | (1%) | 1369 | (0%) | 970 | (0%) |
| Creutzfeldt-Jakob disease | 2 | (0%) | 5 | (0%) | 2 | (0%) |
| Cardioversion | 19 | (0%) | 1 | (0%) | 2 | (0%) |
| Systemic lupus erythematosus | 786 | (0%) | 449 | (0%) | 386 | (0%) |
| Defibrillator insertion | 133 | (0%) | 36 | (0%) | 29 | (0%) |
| Smoker | 127 | (0%) | 939 | (0%) | 484 | (0%) |
| Migraine | 223 | (0%) | 343 | (0%) | 421 | (0%) |
| Down’s syndrome | 57 | (0%) | 55 | (0%) | 70 | (0%) |
| Family history of diabetes | 16557 | (9%) | 72318 | (14%) | 81421 | (15%) |
| Family history of cardiovascular disease | 1328 | (1%) | 6010 | (1%) | 9051 | (2%) |
| **Medication history** |  |  |  |  |  |  |
| Antihypertensive drugs | 79766 | (44%) | 266259 | (51%) | 282635 | (50%) |
| Antidiabetic drugs | 22003 | (12%) | 75510 | (14%) | 78840 | (14%) |
| Antiplatelet drugs | 10968 | (6%) | 29442 | (6%) | 28822 | (5%) |
| Statins | 12088 | (7%) | 25819 | (5%) | 25761 | (5%) |
| Non-steroidal anti-inflammatory drugs | 59805 | (33%) | 199985 | (38%) | 252258 | (45%) |
| Corticosteroids | 53777 | (30%) | 166082 | (32%) | 181784 | (32%) |
| Proton-pump inhibitors | 16737 | (9%) | 38699 | (7%) | 40508 | (7%) |
| H2-receptor antagonists | 45883 | (25%) | 173792 | (33%) | 222804 | (40%) |
| Anticoagulants | 3419 | (2%) | 4048 | (1%) | 4568 | (1%) |
| Antithyroid drugs | 2258 | (1%) | 6493 | (1%) | 7539 | (1%) |
| Nicotine replacement therapy | 859 | (0%) | 1374 | (0%) | 2547 | (0%) |
| Anti-arrhythmic drugs | 1576 | (1%) | 1608 | (0%) | 1610 | (0%) |
| Oestrogen | 3877 | (2%) | 6492 | (1%) | 7071 | (1%) |
| Psychotropic drugs | 25171 | (14%) | 53644 | (10%) | 74513 | (13%) |
| Testosterone | 586 | (0%) | 678 | (0%) | 879 | (0%) |
| Thyroid hormones | 5018 | (3%) | 11045 | (2%) | 12150 | (2%) |
| Fibrates | 3868 | (2%) | 11780 | (2%) | 10914 | (2%) |
| Niacin | 119 | (0%) | 217 | (0%) | 23 | (0%) |
| Cholesterol absorption inhibitors | 157 | (0%) | 43 | (0%) | 67 | (0%) |
| Bile acid sequestrants | 261 | (0%) | 162 | (0%) | 107 | (0%) |
| Omega 3 fatty acids | 95 | (0%) | 10 | (0%) | 2 | (0%) |
| **Clinical laboratory tests** |  |  |  |  |  |  |
| Aspartate transaminase | 24.0 | (19.0-30.0, 11%) | 22.7 | (18.0-30.0, 54%) | 25.0 | (19.0-39.0, 86%) |
| Alanine aminotransferase | 22.0 | (16.0-33.0, 10%) | 20.0 | (15.0-29.0, 4%) | 21.0 | (15.0-30.0, 3%) |
| Hemoglobin A1c | 5.9 | (5.5-6.8, 41%) | 6.0 | (5.6-6.9, 29%) | 6.1 | (5.7-6.8, 30%) |
| Creatine kinase | 103.0 | (68.0-166.0, 57%) | 104.3 | (69.0-167.0, 51%) | 104.0 | (70.0-161.0, 49%) |
| Prothrombin time | 11.6 | (10.9-12.3, 42%) | 11.5 | (10.7-12.3, 40%) | 11.3 | (10.6-12.0, 42%) |
| Low-density lipoprotein cholesterol | 2.8 | (2.3-3.5, 1%) | 3.1 | (2.5-3.7, 1%) | 3.1 | (2.5-3.7, 1%) |
| Potassium | 4.0 | (3.7-4.3, 5%) | 4.2 | (3.9-4.5, 1%) | 4.2 | (3.9-4.5, 0%) |
| Neutrophil | 4.0 | (3.0-5.3, 14%) | 4.2 | (3.2-5.8, 19%) | 4.3 | (3.3-6.0, 18%) |
| Estimated glomerular filtration rate | 83.4 | (69.0-96.7, 32%) | 83.0 | (69.1-96.0, 16%) | 84.0 | (72.0-96.0, 19%) |
| Triglycerides | 1.1 | (0.8-1.6, 0%) | 1.2 | (0.9-1.8, 0%) | 1.2 | (0.9-1.8, 0%) |
| Basophil | 0.0 | (0.0-0.0, 14%) | 0.0 | (0.0-0.0, 20%) | 0.0 | (0.0-0.0, 18%) |
| Blood partial pressure of oxygen | 10.8 | (5.4-14.7, 78%) | 6.7 | (4.0-12.1, 69%) | 6.9 | (4.0-12.1, 74%) |
| Albumin | 43.0 | (40.0-45.0, 10%) | 42.0 | (39.0-44.0, 6%) | 42.8 | (40.0-45.0, 4%) |
| International normalized ratio | 1.0 | (1.0-1.1, 42%) | 1.0 | (1.0-1.1, 40%) | 1.0 | (1.0-1.1, 42%) |
| Diastolic blood pressure | 77.0 | (69.0-86.0, 40%) | 77.0 | (69.0-85.0, 0%) | 78.0 | (70.0-86.0, 0%) |
| Bicarbonate | 24.6 | (21.8-27.3, 74%) | 24.9 | (22.2-27.3, 63%) | 24.7 | (22.0-27.1, 71%) |
| Glucose | 5.2 | (4.8-5.9, 11%) | 5.4 | (5.0-6.2, 1%) | 5.4 | (4.9-6.1, 1%) |
| Erythrocyte sedimentation rate | 28.0 | (15.0-57.0, 68%) | 27.0 | (15.0-50.0, 62%) | 24.0 | (13.0-47.0, 63%) |
| Free thyroxine | 16.2 | (14.3-18.2, 64%) | 13.9 | (12.3-16.0, 67%) | 14.4 | (12.6-16.6, 65%) |
| Troponin I | 0.0 | (0.0-0.0, 85%) | 0.0 | (0.0-0.0, 71%) | 0.0 | (0.0-0.0, 78%) |
| Bilirubin | 9.5 | (7.0-13.0, 10%) | 11.0 | (8.0-14.3, 6%) | 10.8 | (8.0-14.0, 4%) |
| C-reactive protein | 0.4 | (0.3-2.7, 69%) | 0.8 | (0.2-5.1, 61%) | 0.6 | (0.2-3.9, 61%) |
| Total cholesterol | 4.8 | (4.2-5.5, 0%) | 5.2 | (4.5-5.8, 0%) | 5.2 | (4.5-5.9, 0%) |
| Blood pH | 7.4 | (7.4-7.5, 77%) | 7.4 | (7.4-7.4, 67%) | 7.4 | (7.4-7.4, 73%) |
| Systolic blood pressure | 134.0 | (121.0-147.0, 40%) | 136.0 | (123.0-150.0, 0%) | 135.0 | (122.0-149.0, 0%) |
| Thyroid stimulating hormone | 1.4 | (0.9-2.0, 43%) | 1.4 | (0.9-2.1, 28%) | 1.4 | (0.9-2.1, 26%) |
| Lymphocyte | 1.8 | (1.4-2.2, 14%) | 1.8 | (1.4-2.3, 19%) | 1.8 | (1.4-2.3, 18%) |
| Creatinine | 75.0 | (63.0-90.0, 5%) | 74.0 | (62.0-89.0, 1%) | 73.0 | (62.0-87.0, 0%) |
| Platelet | 236.0 | (196.0-282.0, 14%) | 238.0 | (199.0-283.9, 10%) | 239.0 | (200.0-283.0, 9%) |
| Red blood cell | 4.5 | (4.2-4.9, 14%) | 4.5 | (4.2-4.9, 10%) | 4.6 | (4.2-4.9, 9%) |
| Calcium | 2.3 | (2.2-2.4, 34%) | 2.3 | (2.2-2.4, 27%) | 2.3 | (2.2-2.4, 25%) |
| White blood cell | 6.5 | (5.3-8.1, 14%) | 6.8 | (5.6-8.5, 10%) | 6.8 | (5.6-8.4, 9%) |
| Alkaline phosphatase | 69.0 | (57.0-85.0, 10%) | 71.0 | (59.0-86.0, 6%) | 71.0 | (59.0-86.0, 4%) |
| Sodium | 141.0 | (140.0-143.0, 5%) | 141.0 | (139.0-142.0, 1%) | 141.0 | (139.0-142.1, 0%) |
| Eosinophil | 0.1 | (0.1-0.2, 14%) | 0.1 | (0.1-0.2, 19%) | 0.1 | (0.1-0.2, 18%) |
| Hemoglobin | 13.5 | (12.4-14.6, 14%) | 13.5 | (12.5-14.5, 9%) | 13.6 | (12.6-14.6, 9%) |
| Monocyte | 0.4 | (0.3-0.5, 14%) | 0.4 | (0.3-0.6, 19%) | 0.5 | (0.4-0.6, 18%) |
| **General** |  |  |  |  |  |  |
| Average accident and emergency visits per year | 0.1 | (0.0-0.4) | 0.3 | (0.0-0.6) | 0.4 | (0.0-0.6) |
| Average inpatient visits per year | 0.1 | (0.0-0.5) | 0.0 | (0.0-0.5) | 0.0 | (0.0-0.5) |
| Average outpatient visits per year | 3.1 | (0.9-3.8) | 3.9 | (1.6-5.1) | 3.9 | (1.5-4.8) |
| Count of medications | 3.0 | (0.0-5.0) | 4.0 | (3.0-6.0) | 4.0 | (3.0-6.0) |
| All data in n (%), or median (interquartile range), or median (interquartile range, proportion of missing data) unless indicated otherwise. H2 = histamine type 2. *Risk variables in the Kowloon and New Territories cohorts with no significant difference in distribution (p value≥0.05) from the Hong Kong Island (Hong Kong West Cluster) under Chi-square test (categorical risk variables) or in T-test (numerical risk variables). All other risk variables were significant (p value<0.05). | | | | | | |
