# Supplementary material for: Primary prevention cardiovascular disease risk prediction model for contemporary Chinese (1°P-CARDIAC): Model derivation and validation using a hybrid statistical and machine-learning approach
Source: PLoS One. 2025 Jul 28;20(7):e0322419. doi: 10.1371/journal.pone.0322419 (PMC12303301; doi:10.1371/journal.pone.0322419)
Supplement: S7 Table — (DOCX) [file pone.0322419.s011.docx]

| **Supplementary Table 7. Mean (95% CI) of calibration slope on validation cohorts** | | |
| --- | --- | --- |
|  | Kowloon | New Territories |
| 1°P-CARDIAC (full) | 0.98 (0.98, 0.98) | 0.97 (0.97, 0.97) |
| 1°P-CARDIAC (basic) | 1.07 (1.07, 1.07) | 1.08 (1.08, 1.08) |
| PCE (White) | 0.61 (0.61, 0.61) | 0.61 (0.61, 0.61) |
| PCE (African) | 0.56 (0.56, 0.56) | 0.56 (0.56, 0.56) |
| PREDICT | 1.17 (1.17, 1.17) | 1.23 (1.23, 1.23) |
| China-PAR | 0.73 (0.73, 0.73) | 0.73 (0.73, 0.73) |
| Framingham (Asian) | 0.31 (0.31, 0.31) | 0.31 (0.31, 0.31) |
| A measure of model calibration with target value of 1. Values smaller than 1 indicate overfitting, i.e., too low for low-risk patients and/or too high for high-risk patients. Values greater than 1 indicate underfitting, i.e., too high for low-risk patients and/or too low for high-risk patients. CI=confidence interval. Values were measured from 1000 bootstrap replicates. Results of 1°P-CARDIAC (basic), PCE (African), PREDICT, and China-PAR were after recalibration. | | |
