# Supplementary material for: Primary prevention cardiovascular disease risk prediction model for contemporary Chinese (1°P-CARDIAC): Model derivation and validation using a hybrid statistical and machine-learning approach
Source: PLoS One. 2025 Jul 28;20(7):e0322419. doi: 10.1371/journal.pone.0322419 (PMC12303301; doi:10.1371/journal.pone.0322419)
Supplement: S8 Table — (DOCX) [file pone.0322419.s012.docx]

| **Supplementary Table 8. Mean (95% CI) of calibration-in-the-large on validation cohorts** | | |
| --- | --- | --- |
|  | Kowloon | New Territories |
| 1°P-CARDIAC (full) | -0.01 (-0.01, -0.01) | 0.00 (0.00, 0.00) |
| 1°P-CARDIAC (basic) | 0.00 (0.00, 0.00) | 0.00 (0.00, 0.00) |
| PCE (White) | 0.00 (0.00, 0.00) | 0.00 (0.00, 0.00) |
| PCE (African) | 0.05 (0.05, 0.05) | 0.04 (0.04, 0.04) |
| PREDICT | -0.06 (-0.06, -0.06) | -0.05 (-0.05, -0.05) |
| China-PAR | 0.02 (0.02, 0.02) | 0.02 (0.02, 0.02) |
| Framingham (Asian) | 0.12 (0.12, 0.12) | 0.11 (0.11, 0.11) |
| A measure of model calibration with target value of 0. Values greater than 0 means the model overestimates risk in general. Values smaller than 0 means the model underestimates risk in general. CI=confidence interval. Values were measured from 1000 bootstrap replicates. Results of 1°P-CARDIAC (basic), PCE (African), PREDICT, and China-PAR were after recalibration. | | |
