# Supplementary material for: Primary prevention cardiovascular disease risk prediction model for contemporary Chinese (1°P-CARDIAC): Model derivation and validation using a hybrid statistical and machine-learning approach
Source: PLoS One. 2025 Jul 28;20(7):e0322419. doi: 10.1371/journal.pone.0322419 (PMC12303301; doi:10.1371/journal.pone.0322419)
Supplement: S9 Table — (DOCX) [file pone.0322419.s013.docx]

| **Supplementary Table 9. Mean (95% CI) of Harrell's C statistic on validation cohorts before recalibration** | | |
| --- | --- | --- |
|  | Kowloon | New Territories |
| 1°P-CARDIAC (basic) | 0.71 (0.71, 0.71) | 0.72 (0.72, 0.72) |
| PCE (African) | 0.68 (0.68, 0.68) | 0.69 (0.69, 0.69) |
| PREDICT | 0.70 (0.70, 0.70) | 0.71 (0.71, 0.71) |
| China-PAR | 0.69 (0.69, 0.69) | 0.69 (0.69, 0.69) |
| A measure of model discrimination with values ranging from 0.5 to 1, i.e., probability of correct ordering for a randomly selected pair of subjects. CI=confidence interval. Values were measured from 1000 bootstrap replicates. | | |
