# Supplementary material for: Primary prevention cardiovascular disease risk prediction model for contemporary Chinese (1°P-CARDIAC): Model derivation and validation using a hybrid statistical and machine-learning approach
Source: PLoS One. 2025 Jul 28;20(7):e0322419. doi: 10.1371/journal.pone.0322419 (PMC12303301; doi:10.1371/journal.pone.0322419)
Supplement: S10 Table — (DOCX) [file pone.0322419.s014.docx]

| **Supplementary Table 10. Mean (95% CI) of calibration slope on validation cohorts before recalibration** | | |
| --- | --- | --- |
|  | Kowloon | New Territories |
| 1°P-CARDIAC (basic) | 1.26 (1.26, 1.26) | 1.27 (1.27, 1.27) |
| PCE (African) | 0.75 (0.75, 0.75) | 0.73 (0.73, 0.73) |
| PREDICT | 2.49 (2.49, 2.49) | 2.52 (2.52, 2.52) |
| China-PAR | 1.75 (1.75, 1.75) | 1.69 (1.69, 1.69) |
| A measure of model calibration with target value of 1. Values smaller than 1 indicate overfitting, i.e., too low for low-risk patients and/or too high for high-risk patients. Values greater than 1 indicate underfitting, i.e., too high for low-risk patients and/or too low for high-risk patients. CI=confidence interval. Values were measured from 1000 bootstrap replicates. | | |
