# Supplementary material for: Primary prevention cardiovascular disease risk prediction model for contemporary Chinese (1°P-CARDIAC): Model derivation and validation using a hybrid statistical and machine-learning approach
Source: PLoS One. 2025 Jul 28;20(7):e0322419. doi: 10.1371/journal.pone.0322419 (PMC12303301; doi:10.1371/journal.pone.0322419)
Supplement: S11 Table — (DOCX) [file pone.0322419.s015.docx]

| **Supplementary Table 11. Mean (95% CI) of calibration-in-the-large on validation cohorts before recalibration** | | |
| --- | --- | --- |
|  | Kowloon | New Territories |
| 1°P-CARDIAC (basic) | -0.03 (-0.03, -0.03) | -0.02 (-0.02, -0.02) |
| PCE (African) | -0.01 (-0.01, -0.01) | 0.00 (0.00, 0.00) |
| PREDICT | -0.11 (-0.11, -0.11) | -0.10 (-0.10, -0.10) |
| China-PAR | -0.10 (-0.10, -0.10) | -0.08 (-0.08, -0.08) |
| A measure of model calibration with target value of 0. Values greater than 0 means the model overestimates risk in general. Values smaller than 0 means the model underestimates risk in general. CI=confidence interval. Values were measured from 1000 bootstrap replicates. | | |
