# Supplementary material for: Primary prevention cardiovascular disease risk prediction model for contemporary Chinese (1°P-CARDIAC): Model derivation and validation using a hybrid statistical and machine-learning approach
Source: PLoS One. 2025 Jul 28;20(7):e0322419. doi: 10.1371/journal.pone.0322419 (PMC12303301; doi:10.1371/journal.pone.0322419)
Supplement: S12 Table — (DOCX) [file pone.0322419.s016.docx]

| **Supplementary Table 12. Discrimination and calibration performance of 1°P-CARDIAC on derivation cohort** | | | |
| --- | --- | --- | --- |
|  | Harrell's C statistic | Calibration slope | Calibration-in-the-large |
| Basic model | 0.75 (0.75, 0.75) | 0.91 (0.91, 0.91) | 0.00 (0.00, 0.00) |
| Full model | 0.87 (0.87, 0.87) | 0.94 (0.94, 0.94) | 0.00 (0.00, 0.00) |
| All data in n (95% confidence interval). Harrell's C statistic is a measure of model discrimination with values ranging from 0.5 to 1, i.e., probability of correct ordering for a randomly selected pair of subjects. Calibration slope is a measure of model calibration with target value of 1. Values smaller than 1 indicate overfitting, i.e., too low for low-risk patients and/or too high for high-risk patients. Values greater than 1 indicate underfitting, i.e., too high for low-risk patients and/or too low for high-risk patients. Calibration-in-the-large is a measure of model calibration with target value of 0. Values greater than 0 means the model overestimates risk in general. Values smaller than 0 means the model underestimates risk in general. Results were measured from 100 repeats of 10-fold cross validation. | | | |
