# Supplementary material for: Primary prevention cardiovascular disease risk prediction model for contemporary Chinese (1°P-CARDIAC): Model derivation and validation using a hybrid statistical and machine-learning approach
Source: PLoS One. 2025 Jul 28;20(7):e0322419. doi: 10.1371/journal.pone.0322419 (PMC12303301; doi:10.1371/journal.pone.0322419)
Supplement: S13 Table — (DOCX) [file pone.0322419.s017.docx]

| **Supplementary Table 13. Brier score on validation cohorts** | | |
| --- | --- | --- |
|  | Kowloon | New Territories |
| 1°P-CARDIAC (full) | 0.10 (0.10, 0.10) | 0.09 (0.09, 0.09) |
| 1°P-CARDIAC (basic) | 0.12 (0.12, 0.12) | 0.10 (0.10, 0.10) |
| PCE (White) | 0.13 (0.13, 0.13) | 0.11 (0.11, 0.11) |
| PCE (African) | 0.13 (0.13, 0.13) | 0.11 (0.11, 0.11) |
| PREDICT | 0.12 (0.12, 0.12) | 0.10 (0.10, 0.10) |
| China-PAR | 0.12 (0.12, 0.12) | 0.11 (0.11, 0.11) |
| Framingham (Asian) | 0.20 (0.20, 0.20) | 0.18 (0.18, 0.18) |
| A measure of model calibration with target range of 0 to 1. The lower the Brier score is for a set of predictions, the better the predictions are calibrated. Values were measured from 1000 bootstrap replicates. Results of 1°P-CARDIAC (basic), PCE (African), PREDICT, and China-PAR were after recalibration. | | |
