# Supplementary material for: Primary prevention cardiovascular disease risk prediction model for contemporary Chinese (1°P-CARDIAC): Model derivation and validation using a hybrid statistical and machine-learning approach
Source: PLoS One. 2025 Jul 28;20(7):e0322419. doi: 10.1371/journal.pone.0322419 (PMC12303301; doi:10.1371/journal.pone.0322419)
Supplement: S1 Fig — (DOCX) [file pone.0322419.s018.docx]

**Supplementary Figure 1.** **Calibration plots of 1°P-CARDIAC (basic), PCE (African), PREDICT, and China-PAR before recalibration.**
