# Supplementary material for: Primary prevention cardiovascular disease risk prediction model for contemporary Chinese (1°P-CARDIAC): Model derivation and validation using a hybrid statistical and machine-learning approach
Source: PLoS One. 2025 Jul 28;20(7):e0322419. doi: 10.1371/journal.pone.0322419 (PMC12303301; doi:10.1371/journal.pone.0322419)
Supplement: S3 Fig — (DOCX) [file pone.0322419.s020.docx]

**Supplementary Figure 3. Calibration plots for the 1°P-CARDIAC (full) model in the Hong Kong Island (Hong Kong West Cluster) derivation cohort with 95% Confidence interval.** Results were measured from 10-fold cross validation.
