# Supplementary material for: Primary prevention cardiovascular disease risk prediction model for contemporary Chinese (1°P-CARDIAC): Model derivation and validation using a hybrid statistical and machine-learning approach
Source: PLoS One. 2025 Jul 28;20(7):e0322419. doi: 10.1371/journal.pone.0322419 (PMC12303301; doi:10.1371/journal.pone.0322419)
Supplement: S4 Fig — (DOCX) [file pone.0322419.s021.docx]

**Supplementary Figure 4. SHAP (SHapley Additive exPlanations) summary plot of the P-CARDIAC hybrid model​​.** This plot illustrated the direction and magnitude of feature contributions to individual CVD risk predictions. Features were ranked vertically by their mean absolute SHAP value (global importance), with the most influential variables at the top. Top 30 features were visualized. Each point represented a patient, colored by the normalized value of the corresponding feature (red: high, blue: low).
